# Cervical instability in cervical spondylosis patients: Significance of the radiographic index method for evaluation

**DOI:** 10.1007/s00132-018-3635-3

**Published:** 2018-09-25

**Authors:** Mirwais Alizada, Rong Rui Li, Gati Hayatullah

**Affiliations:** Department of Orthopedics-II, First Affiliated Hospital, Jiamusi University, Jiamusi 188 xuefu road, 154000 Jiamusi, Heilongjiang China

**Keywords:** Range of motion, Lordosis, Digital radiography, X-ray film, Comparative study, Bewegungsausmaß, Lordose, Digitale Radiographie, Röntgenfilm, Vergleichende Untersuchung

## Abstract

**Background:**

Cervical spondylosis is one of the most common causes of cervical instability. Various methods are used for measuring cervical instability on X‑ray films. The purpose of this study was to assess the application of the radiographic index method to analyze the radiographic features of cervical spondylosis instability.

**Material and methods:**

Digitized dynamic radiographs of 121 subjects with cervical spondylosis were retrospectively retrieved. The cervical spondylosis patients were divided into two groups according to the symptoms: patients with positive neurological deficits with and without neck symptoms (group I, *n* = 62) and patients with neck symptoms only (group II, *n* = 59). A total of 62 healthy subjects were assigned to the control group (group III). The radiographic indices of cervical curvature, the full flexion to full extension ranges of motion (ROM) and horizontal displacement of the three groups were analyzed and compared with each other.

**Results:**

On flexion-extension views there were significant differences (*p* = 0.00000 [significance of cervical lordosis on flexion view between the three groups], *p* = 0.00271 [significant difference of cervical lordosis between the three groups on extension view]) between the three groups concerning the cervical lordosis: group I had the least cervical curvature, followed by group II and group III. The full flexion to full extension ranges of motion for group I was significantly decreased (*p* *=* 0.0039) when compared with group II and group III. The horizontal displacement at each segmental level (except C2/C3) was significantly higher in group I than that of the other two groups.

**Conclusion:**

With the application of the radiographic index method, cervical spine lordosis, the full flexion to full extension ROM, horizontal displacement, and cervical instability can be accurately illustrated. Cervical spondylosis is an age-related, wear and tear change of the spine that occurs over time. The index of the horizontal displacement ≥0.3 is suggestive of cervical instability.

## Zusammenfassung

**Hintergrund:**

Die zervikale Spondylose ist eine der häufigsten Ursachen für eine zervikale Instabilität. Zur Messung der zervikalen Instabilität auf Röntgenfilmen werden verschiedene Methoden eingesetzt. Der Zweck dieser Studie war es, die Anwendung der radiographischen Index-Methode zur Analyse der radiographischen Merkmale der zervikalen Spondylose-Instabilität zu beurteilen.

**Material und Methoden:**

Digitalisierte dynamische Röntgenaufnahmen von 121 Patienten mit zervikaler Spondylose wurden retrospektiv zusammengestellt. Symptombezogen wurde in 2 Gruppen eingeteilt: Patienten mit positiven neurologischen Defiziten mit und ohne Nackenbeschwerden (Gruppe I, *n* = 62) und Patienten mit Nackenbeschwerden ohne neurologisches Defizit (Gruppe II, *n* = 59). Insgesamt 62 gesunde Probanden bildeten die Kontrollgruppe (Gruppe III). Die radiographischen Indizes – zervikale Krümmung, volle Beugung bis zur vollen Ausdehnung der Bewegungsbereiche („range of motion“, ROM) und horizontale Verschiebung – in den Gruppen wurden analysiert und miteinander verglichen.

**Ergebnisse:**

Bei Flexion-Extension-Aufnahmen gab es bezüglich der zervikalen Lordose signifikante Unterschiede (*p* = 0,00000, *p* = 0,00271) zwischen den 3 Gruppen: in Gruppe I war die zervikale Krümmung am geringsten, gefolgt von Gruppe II und III. Die volle Beugung bis zur vollen Streckung war bei Gruppe I im Vergleich zu den beiden anderen Gruppen signifikant geringer (*p* = 0,0039). Die horizontale Verschiebung auf jeder Segmentebene (außer C2/C3) war in Gruppe I signifikant höher als in den beiden anderen.

**Fazit:**

Mit der Anwendung der radiographischen Indexmethode lassen sich die Halswirbelsäulenlordose, die volle Beugung bis zur vollen Extension (ROM), die horizontale Verschiebung und die zervikale Instabilität genau darstellen. Die zervikale Spondylose ist eine altersbedingte, verschleißbedingte Veränderung der Wirbelsäule, die im Laufe der Zeit auftritt. Ein Index der horizontalen Verschiebung ≥0,3 deutet auf eine zervikale Instabilität hin.

## Introduction

With the increasing incidence of cervical spondylosis, the study methods and measuring modalities of cervical instability are also increasing. The main known method of evaluating cervical instability is the measuring of the horizontal displacement and angular displacement of the cervical vertebral body on lateral X‑ray films; however, so far there is no unified standard for assessment [1–5], thus causing a sense of confusion. Also, due to the existence of impeding factors, such as morphological variability of individual vertebral bodies and radiographic magnification variations, measurement errors between research results are also inevitable. Therefore, to make up for the inadequacies of the previous methods and make research more objective and practical, this study intended to explore a new and more effective X‑ray measurement and evaluation method to analyze cervical instability, the index analysis method.

## Material and methods

All clinical data and digital radiographs of 121 patients with cervical spondylosis, who came to the First Affiliated Hospital of Jiamusi University from 1 October 2015 to 1 October 2017, were collected retrospectively. Diagnosis of cervical spondylosis was made based on the clinical symptoms of the patients (Table 1; [6]). All symptoms of the patients lasted at least 1 month. Cervical spondylosis patients were divided into two main categories based on symptoms: patients with only neck pain and patients with neurological symptoms with/without neck pain. The visual analog scale (VAS) was used to measure the severity of the neck pain. The neck pain was classified as mild (VAS ≤3) or moderate to severe (VAS >3).

**Table 1:** Clinical features of cervical spondylosis

*Symptoms*
Cervical pain aggravated by movement
Referred pain (occiput, between the shoulders blades, upper limbs)
Retro-orbital or temporal pain (from C1 to C2)
Cervical stiffness—reversible or irreversible
Vague numbness, tingling or weakness in upper limbs
Dizziness or vertigo
Poor balance
Rarely syncope, triggers migraine, pseudo-angina
*Signs*
Poorly localized tenderness
Limited range of movement (forward flexion, backward extension, lateral flexion, and rotation to both sides)
Minor neurological changes, e.g. inverted supinator spasms (unless complicated by myelopathy or radiculopathy)

A total of 62 healthy volunteer subjects were recruited. The total number of the study subjects was 183 which were divided into three groups as follow:

1. Patients with positive neurological symptoms with/without neck pain (group I): it comprised 62 cases, 32 males, and 30 females. Ages 24 –74 years, average 43.0 years, 34 cervical radiculopathy cases, 16 cervical myelopathy cases, and 12 mix-type cases.
2. Patients with only neck symptoms (group II): 59 subjects, 13 males, and 46 females. Ages 19 –68 years, average 43.6 years. Exclusion criteria were nondegenerative cervical diseases (trauma, infection, tumor, deformity, inflammation, cancer) or with the history of cervical spine surgery.
3. Normal subjects (group III): 62 normal subjects, 32 males and 30 females, ages 17 –81 years, average 51.2 years.

## Method

### X-ray film analyses

Standard radiographic conditions were applied when taking radiographs as the tube was centered at the C3-C4 intervertebral disc, the distance between the film cassette and x‑ray tube was fixed at 72 inches, and the radiographs were taken without magnification. All lateral digital radiographs of 183 subjects with neutral, flexion and extension views were obtained with the patients all standing erect, their shoulders depressed as far as possible for visualization of the cervicothoracic junction. For a neutral view, the subjects were required to slightly lift the mandible, and look ahead with the line of vision about 15° above the horizontal plane. The requirements for flexion-extension views were full flexion and full extension without applying the external force. The digital X‑ray radiographs were obtained on the PACS II view system (Infinitt, Seoul, Korea) that was used for taking measurements. An orthopedic surgeon, two radiologists, and one statistician assessed this study.

### Measurement items

**Fig. 1:**
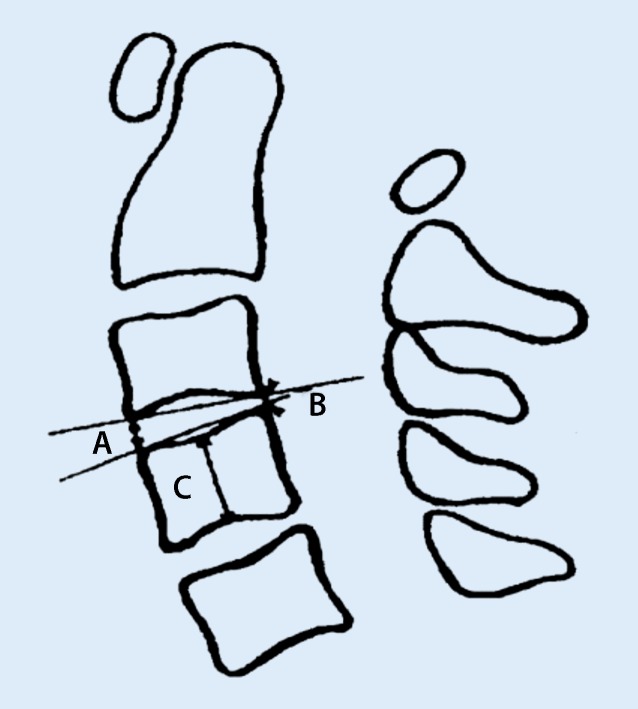
Diagram showing the method for measuring the height of anterior intervertebral space (*A*), height of the posterior intervertebral space (*B*) and height of the center of the subjacent inferior vertebral body (*C*)

**Fig. 2:**
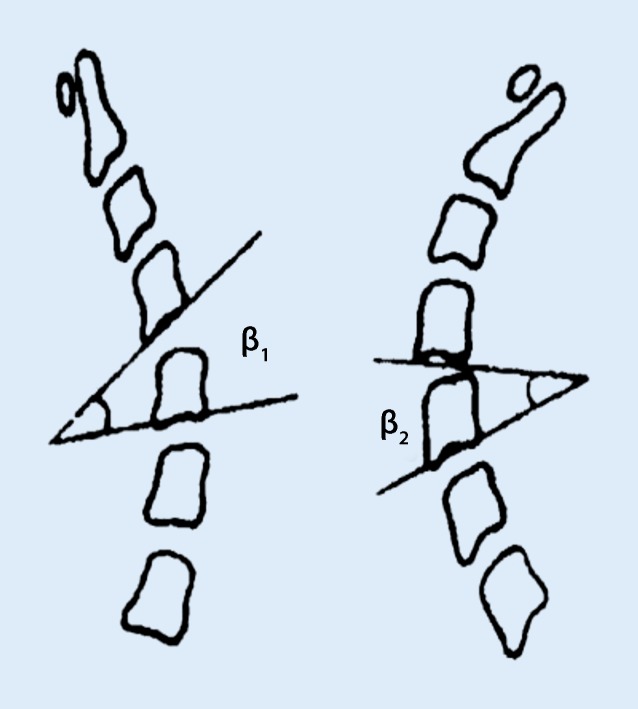
Diagram showing measurement of the angular displacement of the cervical spine. *β1* is the angle on flexion view, *β2* is the angle on extension view

**Fig. 3:**
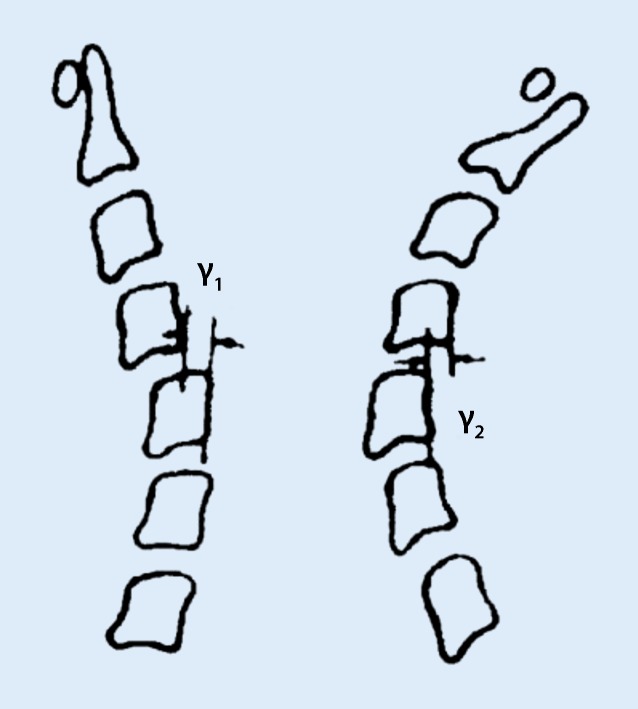
Diagram showing measurement of the horizontal displacement of the cervical spinal vertebrae. *γ1* is the horizintal displacement on flexion view, *γ2* is the horizontal displacement on extension view

Cervical lateral neutral, flexion and extension views were measured separately:

1. Heights of anterior and posterior intervertebral spaces at the level of anterior and posterior vertebral edges (Fig. 1).
2. The central height of each vertebral body: this is the distance measured between the midpoints of the superior and inferior surfaces of the vertebral body (Fig. 1).
3. Vertebral angular displacement: the angle formed at the intersection of the lines drawn parallel to the endplates of subjacent vertebral bodies (Fig. 2).
4. Sagittal diameter of spinal canal**:** it is the smallest sagittal distance measured from the posterior surface of the vertebral body to the closest point of the junction of its laminae and spinous process [7]. In cases of encountering vertebral body posterior margin osteophytes or calcification of ligaments, the minimum distance between the innermost point of the spinal canal and the posterior wall of the spinal canal was considered as the sagittal diameter of the spinal canal.
5. Vertebral horizontal displacement: the perpendicular distance from the line drawn parallel to the posterior-inferior corner to the posterosuperior edge of the superior vertebral body to the line drawn laterally to the posterior margin of the inferior vertebral body (Fig. 3).

### Index method analyses

1. Cervical segmental curvature index: difference between heights of anterior and posterior intervertebral spaces divided by the height of the inferior subjacent vertebral body, i. e. (A – B)/C (Fig. 1).
2. Cervical curvature index: the sum of cervical segmental curvature indexes of cervical spine.
3. The full flexion to full extension range of motion: the difference between cervical curvature indices of flexion and extension views.
4. Horizontal displacement index: absolute value of vertebral horizontal displacement divided by the corresponding sagittal diameter of the spinal canal.
5. Maximum horizontal displacement index: the sum of the horizontal displacement indices of flexion and extension views at each corresponding level.

All measurements were repeated three times by the same observer (HB), each without reference to prior measurements [7]. The average of the distance measurements was rounded up to 0.01 mm, and the average of the angle measurements was rounded up to 1^o^.

## Statistical analyses

The SPSS version 19.0 for Windows (SPSS, Chicago, IL, USA) was used for statistical analyses. Quantitative data are expressed as mean ± SD, one-way ANOVA tests were carried out to compare means of the groups. The Student-Newman-Keuls test was used for multiple comparisons. Data, which did not follow a normal distribution pattern, were expressed as median (lower quartile, upper quartile) {M (Ql, Qu)}. Kruskal-Wallis tests were used for comparison of the groups. Student-Newman-Keuls test was used for multiple comparisons and χ^2^-tests were used for comparison of qualitative data. The partitioning χ^2^ method was used for multiple comparisons. Pearson’s correlation test was performed to analyze the correlation between two variables. It was considered statistically significant when *p* *<* 0.05.

## Results

In the extension view, the cervical curvature of group I has significantly decreased compared to that of group II and group III (*p* = 0.00271) but there is no significant difference between group II and group III. In the flexion view, there are significant differences between three groups (*p* = 0.00000), which are ordered from smallest to largest as, group III, group II and group I; however, in the neutral view there is no significant difference among the three groups (*p* = 0.8415; Table 2).

**Table 2:** Cervical curvature index M (Ql, Qu) between the three groups

	Neutral view	Extension view	Flexion view
Group I	0.72 (0.52, 1.01)	1.11 (0.88, 1.47)	−0.06 (−0.23, 0.15)
Group II	0.72 (0.46, 1.06)	1.42 (1.18, 1.61)^a^	−0.12 (−0.27, −0.04)
Group III	0.82 (0.61, 1.03)	1.37 (1.03, 1.62)^a^	−0.27 (−0.37, −0.16)^a,b^
χ^2^-test	2.26	3.9	−0.45
*P*-value	0.8415	0.00271	0.00000

^a^Comparison with group I

^b^Comparison with group I and group II

The full flexion to full extension ranges of motion of group I decreased significantly more than that of group II and group III (*p* = 0.0039), (Table 3) but there is no significant difference between group II and group III. In group III the C5–6 segment had the maximum range of motion followed by C4–5 and C3–4, although in group I and group II segment C4–5 had the maximum range of motion followed by C5–6 and C3–4.

**Table 3:** Comparison of the full flexion to full extension ranges of motion index (x̄ ± s) between the three groups

	Full flexion to full extension range of motion	No. of patients
Group I	1.19 ± 0.42	62
Group II	1.59 ± 0.49^a^	59
Group III	1.73 ± 0.40^a^	62
F	5.73	–
*P*-value	0.0039	–

^a^Comparison with group I

The three groups were compared at every segmental level with each other (Table 4). The maximum horizontal displacement of group I at the levels of C3 (C3/C4), C4 (C4/C5), C5 (C5/C6), C6 (C6/C7) was significantly higher (*p* = 0.0140, *p* = 0.0004, *p* < 0.0001, *p* < 0.0001) when compared with group II and group III. There was no significant difference between group II and group III.

**Table 4:** Comparison of the maximum horizontal displacement index (x̄ ± s) between the three groups

	Group I	Group II	Group III	F	*P*-value
C2/C3	0.24 ± 0.12	0.20 ± 0.07	0.20 ± 0.07	2.19	0.1170
C3/C4	0.33 ± 0.23	0.25 ± 0.10^a^	0.23 ± 0.09^a^	4.44	0.0140
C4/C5	0.35 ± 0.14	0.25 ± 0.09^a^	0.24 ± 0.13^a^	8.25	0.0004
C5/C6	0.32 ± 0.19	0.20 ± 0.10^a^	0.18 ± 0.09^a^	10.56	<0.0001
C6/C7	0.33 ± 0.19	0.17 ± 0.08^a^	0.18 ± 0.07^a^	10.90	<0.0000

$\overline{x}$ mean of sample, *s* standard deviation of the sample

^a^Comparison with group I

Within every group the maximum horizontal displacement indices at motion segmental level (Table 5) were compared. Among group I, the maximum horizontal displacement was most common in C4 (C4/C5; *p* = 0.017176). In group II, the maximum horizontal displacement was most common in C3 (C3/C4) and C4 (C4/C5). Secondarily, it was most common in C5 (C5/C6) and C6 (C6/C7; *p* = 0.000123). In group III there was no statistically significant difference among the various segments.

**Table 5:** Comparison of the maximum horizontal displacement index M (Ql, Qu) between the three groups

	Group I	Group II	Group III
C2-C3	0.19 (0.16, 0.27)	0.17 (0.14, 0.25)	0.17 (0.15, 0.25)
C3-C4	0.26 (0.22, 0.29)	0.24 (0.18, 0.31)^a^	0.21 (0.16, 0.28)
C4-C5	0.33 (0.25, 0.44)^a^	0.23 (0.17, 0.32)^a^	0.21 (0.15, 0.29)
C5-C6	0.24 (0.19, 0.46)	0.17 (0.14, 0.25)^b,c^	0.15 (0.13, 0.22)
C6-C7	0.24 (0.18, 0.49)	0.15 (0.13, 0.21)^b,c^	0.18 (0.12, 0.20)
χ^2^-test	12.0237	23.0617	13.8195
*P*-value	0.017176	0.000123	0.007894

*M (Ql, Qu)* median (lower quartile, upper quartile)

^a^Comparison with C2-C3

^b^Comparison with C3-C4

^c^Comparison with C4-C5

In the flexion-extension views the incidence of the horizontally displaced segments among the three groups was significantly different with orientation of highest to lowest as group II, group I, and group III, (χ^2^ = 50.8221, df = 2, *p* < 0.0001), while in the neutral view there was no significant difference (χ^2^ = 2.512, df = 2, *p* = 0.2847) among the three groups (Table 6).

**Table 6:** Incidence of horizontally displaced segments between the three groups

	Neutral view		Flexion-extension view	
	Segment no.	Incidence (%)	Segment no.	Incidence (%)
Group I	72	23.23	159	25.65
Group II	57	19.32	264^a^	44.75
Group III	57	18.39	243^a,b^	39.19
χ^2^-test	2.512		50.8221	
df	2		2	
*P-value*	0.2847		<0.0001	

*df* degrees of freedom

^a^Comparison with Group I

^b^Comparison with group II

The number of maximum horizontal displaced segments between the three groups was significantly different for each of the three categories, i. e. horizontal displacement index of ≥0.20, ≥0.25, and ≥0.30, respectively. The three groups in descending order according to the number of maximally displaced segments are group I, group II and group III (χ^2^ = 18.4280, 42.4038, 37.2469; *p* ≤ 0.0001,* p* < 0.0001,* p* < 0.0001, respectively; Table 7).

**Table 7:** Numbers of maximum horizontal displaced segments according to the three categories

	≥0.20	≥0.25	≥0.30
Group I	148	99	51
Group II	100	46	15
Group III	87^a,b^	32^a,b^	11^a,b^
χ^2^-test	18.4280	42.4038	37.2469
*P*-value	<0.0001	<0.0001	<0.0001

^a^Comparison with group I

^b^Comparison with group II

**Fig. 4:**
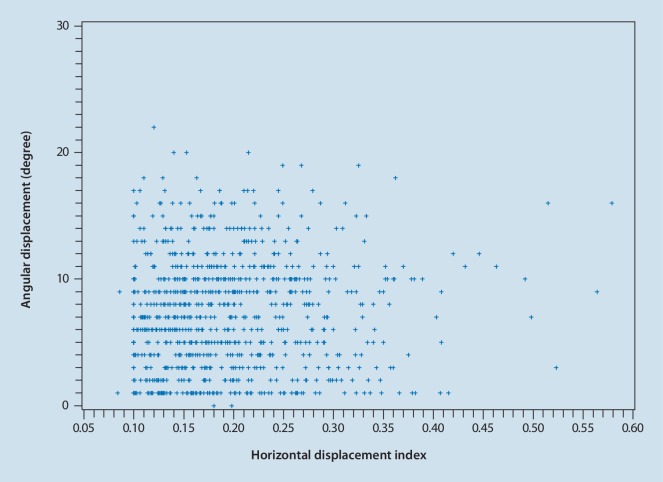
Correlation between the angular and horizontal displacements of the cervical spine

There was no correlation between horizontal displacement and angular displacement (r = 0.3384, *p* = 0.3242), as shown in (Fig. 4).

## Discussion

### Significance of radiographic index analysis method

There are different perceptions and values for determining spinal instability: cervical instability is considered present if the horizontal displacement of cervical vertebra is more than 2 mm, 3 mm or 3.5 mm (depending on the study) as well as 20% of the sagittal vertebral diameter, or if the angular displacement is more than 10°, 11°, and 12°. Schlicke et al. [8] defined spinal instability when the translation motion on sagittal x‑ray was >1.7 mm or >5.7^o^ change in the intervertebral angular movement. Some authors reported that the horizontal and angular displacements have different standards on neutral view and flexion-extension views [3, 5]. Different cervical vertebral positions, individual vertebral body variance and different radiographic magnifications result in different X‑ray images, thus producing various measured values [9]. At the same time, the values in many studies are obtained from the conversion of actual values or magnification values [7, 10]. It is crucial to accurately determine the posterior edge of the vertebral body without the effects of osteophyte and ligament calcification for measuring the sagittal diameter of the spinal canal; otherwise, it will severely affect the results. The radiographic index analysis method eliminates the interference of the main influencing factors, such as individual vertebral body difference and radiographic magnification variation. Moreover, the suspicion about the authenticity of the result, which has been drawn by conversion of the actual value obtained, has also been eliminated and conversion of values is not necessary anymore. The values obtained via the index method are directly used as the result. Spinal instability often leads to spinal stenosis and the spondylotic changes lead to diffuse and focal axonal changes. The diameter of the spinal canal is crucial for the normal function of the spinal cord [11].

The index method has a preponderance for the depiction of cervical spine instability. This method to a large extent determines the impact of vertebral displacement on spinal canal tissues and measures the absolute value of the spinal canal diameter. The relative displacement of the vertebral body, which causes the spinal stenosis particularly in cases of a vertebral body posterior edge osteophyte, will cause the spinal cord to be pinched between the pincers of posteroinferior margin of the superior vertebral body and the anterosuperior margin of the lamina of the inferior vertebra (pincers mechanism; [11]).

Developmental spinal stenosis is a static factor of spinal cord compression and vertebral instability as a dynamic factor exacerbates this injury [12]. Dynamic spinal stenosis is directly related to two factors: the original sagittal diameter of the spinal canal and vertebral body displacement. Naturally, the more significant the vertebral displacement the smaller the dynamic sagittal diameter of the spinal canal and the more damage to the spinal cord. Therefore, to reflect the effect of vertebral horizontal displacement on the spinal canal tissues more accurately, the two main factors that affect spinal cord injury are expressed simultaneously. The index analysis method is more favorable than the technique of merely measuring horizontal displacement distance.

The application of the index method also provides a new means for efficiently evaluating cervical curvature and flexion-extension range of motion. Previous methods described the arc of the cervical spine by the use of the height (D value) of the posterior margin or anterior margin of the cervical spine as well as the angle formed between the lines parallel to the posterior edges or inferior endplates of C2 and C7 vertebral bodies [13]. It reflects the curvature of the cervical spine as a whole, but when encountering irregular changes in cervical spine curvature, such as a double arc, upper arc and lower straightened or upper straightened and lower arc cervical spine, then this method is not sufficient in such abnormal situations of intervertebral activity. The index method can describe the cervical spine as a whole and also displays the changes between various vertebral bodies, gives a more specific description about the cervical curvature and range of motion (ROM) and furthermore can compare the differences between each vertebral body (subjacent vertebral bodies).

### Cervical instability and cervical spondylosis

**Fig. 5:**
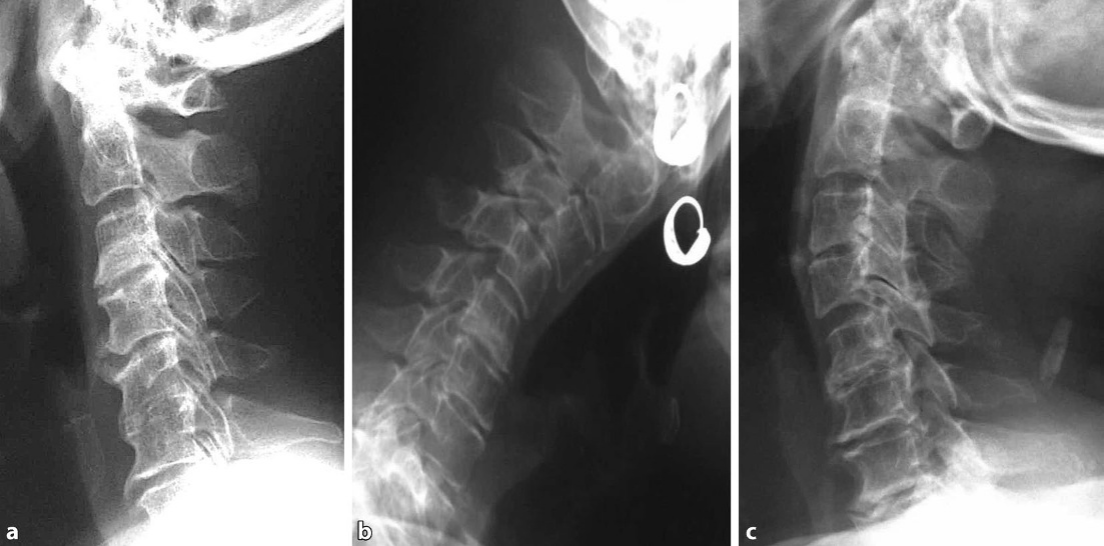
Cervical spine dynamic changes of the same patient with cervical spondylosis. **a** Neutral position: cervical hypolordosis with slight reverse in cervical curvature (kyphosis). The C5-C6 intervertebral space disappeared due to the bridging of C5 and C6 vertebral bodies. The C3-C4 and C4-C5 intervertebral spaces are slightly narrowed with a C6 vertebral anteroinferior edge claw osteophyte. **b** Flexion position: obvious anterolisthesis of C3 over C4. **c** Extension position: hypolordosis with a noticeable decrease in motion. Slight anterolisthesis of C4 over C5, severe narrowing of C5-C6 intervertebral space

Cervical spine degenerative changes are one of the most common causes of cervical instability. There are authors [3] who have studied it as a separate disease. This study found that group I had a significantly higher number of maximum horizontal displacement segments with a displacement index of ≥0.30 when compared to group II and group III. In flexion-extension views, the cervical curvature indexes of group I were significantly lower than that of group II and group III. The spines appeared hypolordotic, straightened or even kyphotic. Furthermore, the full flexion to full extension ROM of group I was significantly smaller than the other two groups. Thus, it indicates the concomitant changes of decreased cervical curvature, the full flexion to full extension ROM, and increased intervertebral displacement in the process of cervical spine degeneration (Fig. 5). Therefore, the authors believe that it is more appropriate to consider cervical instability as a stage of the cervical degeneration process. During this stage, the overall cervical motion and intervertebral segmental movement show abnormal pathological changes.

The maximum horizontal displacement of group I was more significant when compared with group II and group III at all segmental levels except C2-C3. The C4-C5 level had the largest maximum horizontal displacement among group I. In group II the C3-C4 and C4-C5 (with no significant difference between them) levels had the largest displacement distance. There was no significant difference between group II and group III. These results are consistent with those in the literature [14, 15].

In group III with each intervertebral flexion-extension, C5–C6 has the maximum ROM followed by C4–C5 and C3–C4 but among group I and group II C4–5 had the maximum ROM secondarily followed by C5–6 and C3–4. The authors believe that C4–5 and C5–C6 intervertebral spaces are located at the center of the cervical spine. It is the site of maximum stress concentration, with maximum ROM, high frequency of activity, and most prone to chronic inflammatory injury. Hence, cervical degeneration and even cervical instability take place quickly. These results concur with the literature [16].

There are two variables, namely the absolute value of vertebral horizontal displacement and the sagittal diameter of the spinal canal, which crucially affect the horizontal displacement index. In this study, the horizontal displacement index values of various segments of group III were different; therefore, it is not yet possible to define a standard uniform value. Here a reference value is proposed based on the maximum horizontal displacement index of maximally occurring segments, i.e. C4-C5 and C5-C6 and postulate cervical spine instability when the maximum horizontal displacement index ≥0.30. According to this criterion, the numbers of unstable segments detected in group I, group II and group III were 51, 15 and 11, respectively with group I having the significantly highest number (*p* < 0.0001).

The horizontal displacement index of the vertebral body and the angular displacement showed no correlation (r = 0.3384, *p* = 0.3242), indicating that if the horizontal displacement index of a vertebral body increases, it is not necessary that the vertebral angular displacement should also increase. Some parts of larger angular displacement were found without horizontal displacement. Therefore, it is believed that in assessing vertebral instability, both are essential but neither correlates nor is parallel to each other.

According to the ages of group I and group II, the former peak was located between 50 and 59 years old while the latter peak was located between 40 and 49 years old. The peak in group II was 10 years earlier than group I, suggesting that neck symptoms persist for a quite extended period before the cervical spondylosis occurs, which is consistent with the literature [14]. It indicates that cervical degeneration is a long and gradual process.

Comparing the three groups, in flexion-extension views the incidence of horizontally displaced segments was significantly different ranging from highest to lowest as group II, group III and group I. Considering the maximum horizontal displacement distance, there was no significant difference between group II and group III.

In the neutral view there was no significant difference among the groups concerning cervical curvature; however, in the flexion view group I and group II showed significantly decreased curvature (with no significant difference between them) when compared to group III. In the extension view there was no significant difference between the cervical curvatures of group II and group III but both of them showed significantly increased cervical curvature when compared with group I. The full flexion to the full extension ROM of group I significantly decreased compared to other two groups, while the difference between group II and group III was not significant. At the same time correlating the age distribution of each group, it was concluded that the degeneration process, cervical displacement, and even cervical instability took place earlier than that of the decrease in the flexion-extension ROM. At this point cervical lordosis has already decreased but this decrease was slighter when compared to the cervical spondylosis stage. Cervical instability co-occurred and/or was followed by gradual reduction of cervical lordosis while the full flexion to full extension ROM decrease took place at the end.

Every study has limitations and so does this study.

1. Although the X‑ray technicians positioned all the subjects in the same manner as per the protocol of the hospital, the accuracy of our data cannot be guaranteed with 100% assurance.
2. A prospective study may give different results when compared to this retrospective investigation. As this is a radiographic study the results might not be significantly different from that of a prospective study.
3. It is necessary to clinically correlate the patients and test the usefulness of the radiographic index method in cervical spondylosis patients with cervical instability.

Despite these drawbacks, to our knowledge this study is the first of its kind, which suggests a new radiographic index method for evaluating degenerative cervical instability.

## Abbreviations

CR: Computed radiography
DR: Digital radiography
PACS: Picture archiving and computing system
ROM: Range of motion
VAS: Visual analog scale
